# Intraoperative adverse events and management strategies in laparoscopic enhanced-view totally extraperitoneal repair (eTEP): a guide to safe introduction

**DOI:** 10.1007/s10029-026-03775-8

**Published:** 2026-07-03

**Authors:** Jörg Filser, Isabelle Obrecht, Daniel C. Steinemann, Fiorenzo Angehrn, Michael Meir, Christian Jurowich, Christoph-Thomas Germer, Beat P. Müller, Julian Süsstrunk, Johannes Baur

**Affiliations:** 1 Department of General, Visceral and Oncologic Surgery, Innklinikum Altötting, Altötting, Germany; 2 Department of Visceral Surgery, University Digestive Health Care Center, St. Clara Hospital and University Hospital Basel, Basel, Switzerland; 3 Department of General, Visceral, Transplantation, Vascular and Pediatric Surgery, University Hospital Würzburg, Würzburg, Germany

**Keywords:** Ventral hernia, Laparoscopic hernia repair, eTEP, Intraoperative complications

## Abstract

**Background:**

Enhanced-view totally extraperitoneal repair (eTEP) is a promising minimally-invasive technique for ventral hernia repair, allowing extensive retromuscular dissection and large mesh placement with low rates of wound complications and a fast recovery. The learning curve, however, is substantial and technical expertise is crucial for satisfying outcomes. The aim of this study is to critically analyse intraoperative adverse events and provide guidance for safe introduction of eTEP.

**Materials and methods:**

All patients undergoing eTEP for primary or incisional ventral hernias between October 2023 and December 2024 at three centres were included in this prospective study. eTEP was divided in 10 surgical steps and each was rated for its difficulty (1–5 points) and analysed for the incidence of adverse events. Surgeon workload was measured using the NASA-Task-Load-Index (0-100) and perioperative outcomes were reported.

**Results:**

A total of 125 patients with a median BMI of 31 kg/m^2^ and 30% female sex were included. Dissection of the hernia and steps involving suturing of the hernia orifice or posterior defects were rated as most difficult. The most common minor adverse events were peritoneal injury during hernia dissection (52%) and minor bleeding during rectus sheath dissection (up to 45%). Critical intraoperative events were common and included injury to the linea alba (6.4%), major bleeding during posterior rectus sheath dissection (up to 4.8%), conversion to open surgery (2.4%) or hybrid procedure (5.6%). Median operation time was 92 min, median length of stay was 2 days. Surgical revisions were necessary in 3.4%. Median overall procedural workload using the NASA raw score was 39.5/100, strongly correlating with operative time.

**Conclusion:**

Hernia dissection and defect closure in laparoscopic eTEP are particularly technically challenging and associated with a high surgeon workload. Due to its technical complexity and frequent intraoperative adverse events, laparoscopic eTEP should be adopted within structured training pathways and appropriate case selection.

**Supplementary Information:**

The online version contains supplementary material available at 10.1007/s10029-026-03775-8.

## Introduction

Surgical repair is the only durable cure for primary and incisional ventral hernia and represents one of the most commonly performed procedures in general surgery. There is a large variety of procedures used worldwide, with robust data showing that minimally-invasive repair techniques lead to less wound complications and hernia recurrences compared to open repair [1]. 

The field of minimally-invasive techniques is currently rapidly expanding [2]. Amongst the newly introduced techniques, Enhanced View Totally Extraperitoneal Repair (eTEP), first described in 2012 by J. Daes, is of particular interest [3]. This technique allows an extensive retromuscular dissection and thus insertion of large meshes, it can be performed laparoscopically or robotically and may be extended with posterior component separation in large incisional hernia repair [3, 4]. In addition, it brings all the advantages of a minimally-invasive approach, particularly reduction of surgical site infections and postoperative pain with a faster recovery [5]. 

It is, however, a technically challenging procedure, and the learning curve is substantial, in both the laparoscopic and the robotic approach [6, 7]. Since this procedure is relatively new, the literature does not provide ample data on adverse events or intraoperative difficulties. Thus, the aim of this study is to analyze intraoperative complications and adverse events in laparoscopic eTEP and report on surgeon workload and subjective difficulty of the ten crucial steps in this procedure. Furthermore, the present study offers suggestions how to deal with intraoperative complications and technical difficulties and may be used as a guide for a safe introduction of eTEP.

## Methods

### Study design

This prospective observational study utilized patient data from the CROSSFIRE database (MultiCentre InteRnational PrOSpective DatabaSe For Ventral HernIa REpair). All patients who underwent laparoscopic eTEP repair for epigastric or umbilical hernias between October 2023 and December 2024 were included. The study protocol and data analysis were approved by the local ethics committees (EKNZ 2024 − 01463 and 108/24). All patients provided written informed consent prior to participation.

### Inclusion and exclusion criteria

All patients aged ≥ 18 years who underwent eTEP repair at participating centers and had provided signed consent for inclusion in the CROSSFIRE database were eligible and consecutively included. Patients who underwent concomitant surgical interventions such as inguinal hernia repair were excluded.

### Operative technique: 10 steps in eTEP

The surgical procedure was performed as previously described in detail [8, 9]. The procedure was divided in ten critical procedural steps as described by Morrell et al.:

- Step 1: Access for first trocar: Following preoperative preparation and patient positioning, a 12 mm camera trocar is inserted near the left costal margin under direct vision. CO₂ insufflation aids in expanding the retromuscular space. Alternatively, the trocar can be placed in open technique.
- Step 2: Dissection of the ipsilateral rectus sheath: The laparoscope is used for blunt dissection to enlarge the space, visualize the epigastric vessels and facilitate insertion of two additional working ports.
- Step 3: Establishment of working trocars: Once adequate space is achieved, two working trocars are inserted under visual control. These ports are placed laterally and caudally along the midclavicular line, yet medial to the semilunar line to remain within the retro-rectus space.
- Step 4: Incision of the posterior rectus sheath of the access side: The medial border of the posterior sheath is incised carefully to preserve the linea alba. Cranial cross-over is performed, as the falciform ligament provides a safe zone to enter the pre-peritoneal space.
- Step 5: Incision of the contralateral posterior rectus sheath: Upon visualization of the contralateral posterior rectus sheath, an incision is made to gain access to the right retromuscular space. The supraumbilical linea alba is explored, especially in the presence of diastasis recti, to exclude concomitant hernias.
- Step 6: Dissection of the rectus sheath contralateral to the access side: The contralateral posterior rectus sheath is dissected to gain space and provide sufficient room for lateral mesh placement and
- Step 7: Dissection of the hernia, hernia sac and hernia defect: The hernia sac is carefully dissected, with minimal use of diathermy, particularly in the presence of bowel or umbilical skin, to avoid thermal injury. The hernia sac is separated from the surrounding fascia and reduced.
- Step 8: Suturing of the posterior layer (if necessary): Defects in the posterior rectus sheath or peritoneum are closed to prevent direct contact between bowel and mesh, typically using absorbable sutures or barbed sutures for larger defects.
- Step 9: Suturing of the hernia defect: The hernia orifice is closed using slowly absorbable sutures. Lowering the insufflation pressure facilitates approximation of the fascial edges.
- Step 10: Mesh placement: The mesh size is based on the hernia dimension and available space. The retromuscular space is measured intracorporally for more accuracy, and a non-absorbable mesh is tailored accordingly. Fixation with glue or sutures is optional.

### Data acquisition

Baseline patient characteristics - including BMI, Charlson Comorbidity Index (CCI), and risk factors - were collected alongside perioperative outcomes such as mesh-to-defect ratio (calculated as mesh area in cm^2^ divided by defect area in cm^2^), operative time, and postoperative pain (VAS 0–10). Each procedural step was individually rated by the surgeon for technical difficulty using a Numeric Rating Scale (NRS 1–5; 1 = very easy, 5 = most difficult) and screened for step-specific intraoperative adverse events. The overall surgeon workload for the entire procedure was quantified using the NASA-Task-Load Index (NASA-TLX, 0–100) [10]. NASA-TLX Raw Score was calculated for each procedure as the arithmetic mean of its six individual dimensions. Critical intraoperative events were defined as linea alba injury, conversion to open or hybrid surgery, or major bleeding (defined as bleeding that needed additional efforts to control such as clips or hemostatic agents). Hybrid surgery was defined as a procedure, where hernia and mesh area dissection were performed minimally-invasive but closure of posterior and/or anterior defects were performed in an open technique. Postoperative complications were classified according to Clavien–Dindo and the Comprehensive Complication Index (CCI) [11]. Statistical analysis was performed using R-Studio, presenting continuous variables as mean (± SD) or median [IQR] based on Shapiro-Wilk normality testing. To identify significant differences in difficulty between the ten steps, a global Kruskal-Wallis test was followed by post-hoc pairwise Wilcoxon signed-rank tests with Holm-Bonferroni correction. The magnitude of these differences was quantified by the effect size, interpreted as small (0.10), medium (0.30), or large (≥ 0.50). The correlation between these workload scores (NASA-TLX Raw score) and the recorded operative times was then assessed using Pearson’s correlation coefficient.

## Results

### Patient demographics

A total of 125 patients underwent the eTEP between October 2023 and December 2024. The median body mass index (BMI) was 30.9 kg/m^2^ (IQR 26.8, 34.1) and 29.6% of patients were female. The median Charlson Comorbidity Index was 2 (IQR 0, 3) and 16.8% suffered from type 2 diabetes mellitus. A detailed description of baseline characteristics is shown in Table 1.

**Table 1:** Baseline characteristics

	N = 125^1^
Female sex	37 (30%)
Age at surgery	55 (42, 66)
BMI (kg/m^2^)	31 (27, 34)
Obesity classification	
No Obesity (BMI 25 kg/m^2^ or less)	21 (17%)
WHO Pre-Obesity (BMI 25.0-29.9 kg/m^2^)	35 (28%)
WHO Class 1 (BMI 30.0-34.9 kg/m^2^)	41 (33%)
WHO Class 2 (BMI 35.0-39.9 kg/m^2^)	14 (11%)
WHO Class 3 (BMI 40 kg/m^2^ or more)	14 (11%)
Charlson Morbidity Index	2.00 (0.00, 3.00)
Diabetes mellitus	21 (17%)
Hypertension	38 (30%)
Active smoking	31 (25%)
Platelet aggregation inhibitors	15 (12%)
Therapeutic anticoagulation	11 (9.2%)

^1^ n (%); Median (Q1, Q3)

*BMI* body mass index, *WHO* World Health Organisation

### Procedural details and perioperative outcome

Median hernia diameter was 2 cm (IQR 2, 3) and 31.2% were incisional hernias. Median operative time was 92 min (IQR 73, 110), median mesh area was 300cm^2^ (IQR 300–352) and median mesh-defect-ratio was 78cm^2^ (IQR 37, 115). Seven patients (5.6%) underwent a hybrid procedure. Median length of stay was 2 days (IQR 1, 2) and operative revision was performed in 4 patients (3.2%). Follow-up duration in the present report was 6 weeks and completed in all patients. Detailed information on procedural data and postoperative outcomes is displayed in Table 2.

**Table 2:** Procedural details

	N = 125^1^
Hernia type	
Primary	86 (69%)
Incisional	39 (31%)
EHS classification primary hernias	
small (< 2cm)	18 (21%)
medium (2 - 4cm)	63 (73%)
large (> 4cm)	5 (5.8%)
EHS classification incisional hernias (location)	
M1	4 (10%)
M2	21 (54%)
M3	12 (31%)
M4	2 (5.1%)
EHS classification incisional hernias (width)	
W1 (< 4cm)	26 (67%)
W2 (4 - 10cm)	12 (31%)
W3 (> 10cm)	1 (2.6%)
Cut-suture-time [min]	92 (73, 110)
Hernia diameter [cm]	2.00 (2.00, 3.00)
Defect area [cm^2^]	5 (3, 7)
Mesh area [cm^2^]	300 (300, 352)
Mesh-defect-ratio	78 (37, 115)
Diastasis recti	31 (33%)
Concomitant hernia	42 (34%)
Mesh fixation	
None	105 (85%)
Glue	18 (15%)
Suture, non-resorbable	1 (0.8%)
Length of stay (days)	2.00 (1.00, 2.00)
Pain after 2 days [VAS]	3.00 (2.00, 4.00)
Pain after 6 weeks [VAS]	1.00 (1.00, 3.00)
Surgical revision	4 (3.2%)
Comprehensive Complication Index	0 (0, 0)

^1^ n (%): Median (Q1, Q3)

Mesh-Defect Ratio is Mesh Area / Defect Area.

*EHS* European Hernia Society, *VAS* visual analogue scale. Mesh-defect-ratio calculated as mesh area in cm^2^ divided by defect area in cm^2^

Median operative time and hernia diameter in the first 50% of patients was 89 min and 2 cm and 94 min and 2.5 cm in the second 50% of patients, respectively (*p* = 0.6 and 0.06) indicating that the learning curve was likely surpassed before trial start.

### Technical difficulties and surgeon workload

**Fig. 1:**
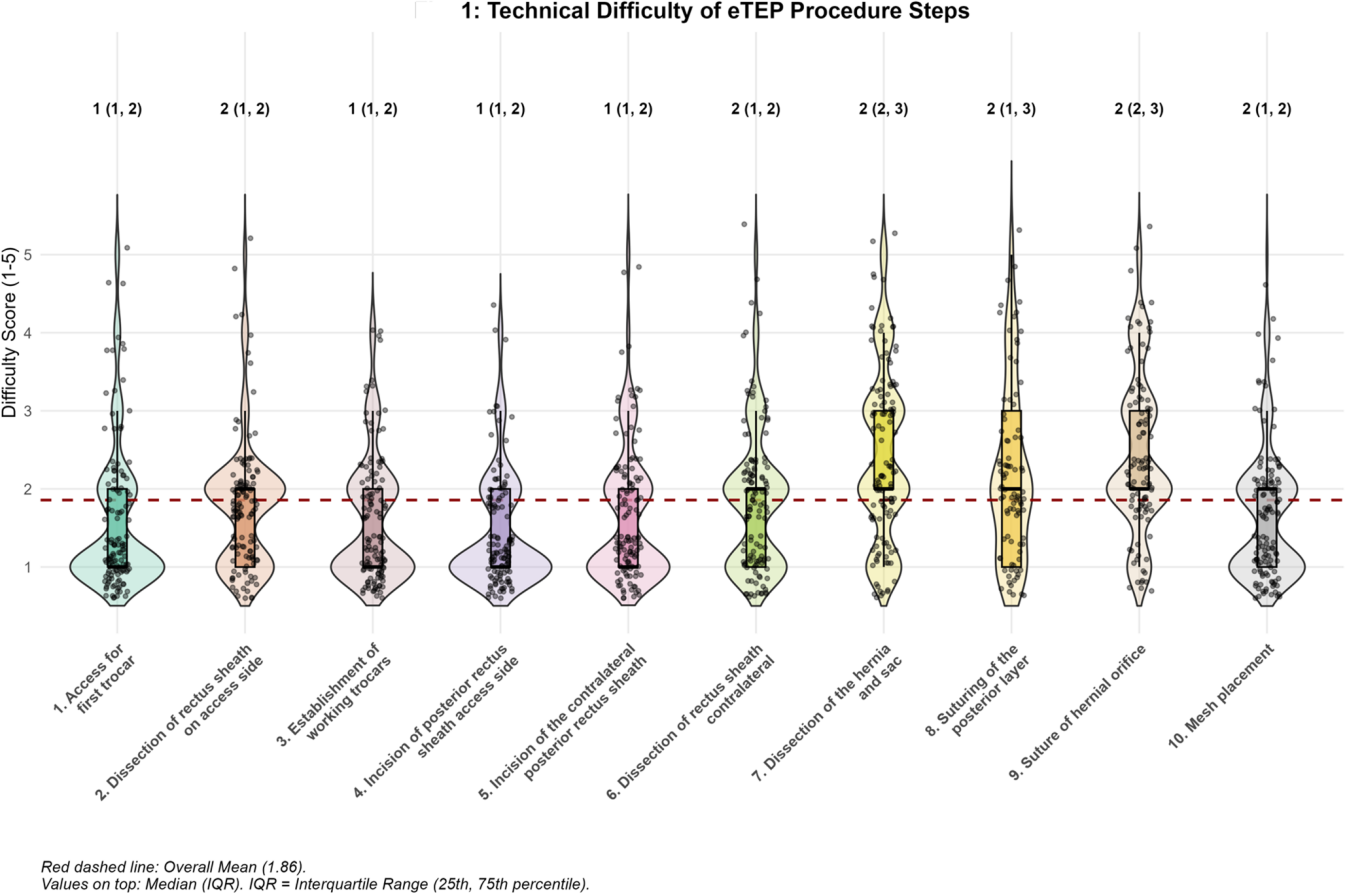
Technical difficulty of enhanced-view totally extraperitoneal repair (eTEP) procedure steps

The overall surgical procedure was rated with a mean difficulty score of 1.88 out of 5. The most challenging technical steps were the dissection of the hernia, hernia sac and hernia defect (step 7), the suture of the posterior layer (step 8) and the suture of the hernia defect (step 9) which were rated as 2/5 (IQR 2, 3), 2/5 (IQR 1, 3) and 2/5 (IQR 2, 3). Figure 1 displays the technical difficulties of each procedural step as a violin plot. In addition, Table S1 (supplementary material) provides a comparative analysis of technical difficulty levels, indicating that step 7 and step 9 were significantly more difficult compared to most other procedural steps.

Injury to the linea alba occurred in 8 patients, mainly during step 5 (3.2%) and step 7 (2.4%). Conversion to open surgery occurred during step 2 (1.6%) and step 4 (0.8%). Major bleeding occurred during the dissection of the posterior rectus sheath on the access side (step 2, 1.6%) and on the contralateral side (step 6, 4.8%). Conversion to a hybrid procedure occurred mainly in step 7 (2.4%) and step 9 (2.4%). The cumulative incidence of critical intraoperative events was 20.8%, where multiple events may have occurred in one patient.

The most seen minor intraoperative events were injury to the peritoneum during the dissection of the hernia (step 7, 52%) and minor bleeding during dissection of the rectus sheaths (step 2, 45% and step 6, 43%). Table 3 provides detailed information on intraoperative adverse events for all procedural steps.

**Fig. 2:**
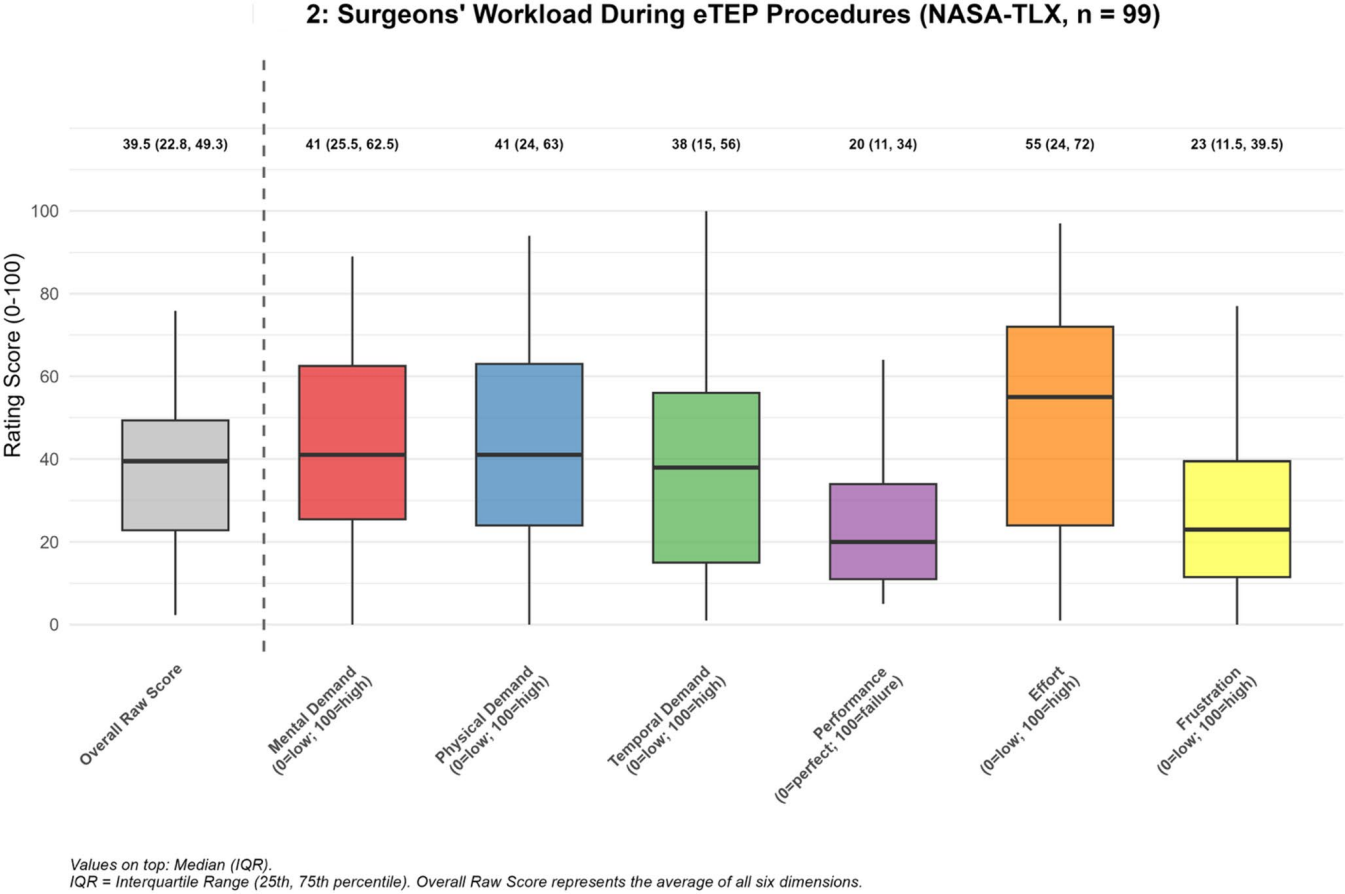
Surgeons’ workload during eTEP procedure (NASA-TLX)

The NASA-TLX questionnaire was completed after 99 (79.2%) surgeries. Median overall procedural workload using the NASA raw score was 39.5/100 (IQR 22.8, 49.3). Detailed information on NASA-TLX sub-scores is shown in Fig. 2, indicating that eTEP is associated with a high surgeons’ effort scoring 55/100 points (IQR 22, 74). There is a strong correlation between NASA-TLX raw score and OR time with an R value of 0.67 (*p* < 0.0001) as shown in Figure S1 (supplementary material).

**Table 3:** Intraoperative adverse events

	N = 125^1^
Access for first trocar	
Minor bleeding	9 (7.2%)
Pneumoperitoneum	7 (5.6%)
Penetration of posterior rectus sheath	3 (2.4%)
Dissection of rectus sheath on access side	
Minor bleeding	56 (45%)
Pneumoperitoneum	4 (3.2%)
Major bleeding	2 (1.6%)
Conversion to open surgery	2 (1.6%)
Establishment of working trocars	
Minor bleeding	26 (21%)
Injury to posterior sheath/pneumoperitoneum	3 (2.4%)
Incision of posterior rectus sheath access side	
Minor bleeding	20 (16%)
Injury to peritoneum/pneumoperitoneum	11 (8.8%)
Conversion to open surgery	1 (0.8%)
Injury to Linea alba	1 (0.8%)
Incision of the contralateral posterior rectus sheath	
Minor bleeding	26 (21%)
Difficulty in finding posterior rectus sheath	10 (8.0%)
Injury to Linea alba	4 (3.2%)
Dissection of posterior rectus sheath contralaterl of the access side	
Minor bleeding	54 (43%)
Injury to peritoneum/pneumoperitoneum	10 (8.0%)
Major bleeding	6 (4.8%)
Dissection of the hernia, hernia defect and hernia sac	
Injury to peritoneum/pneumoperitoneum	65 (52%)
Minor bleeding	43 (34%)
Injury to Linea alba	3 (2.4%)
Conversion to hybrid surgery	3 (2.4%)
Injury to umbilical skin	2 (1.6%)
Suturing of the posterior layer (if necessary)	
Minor bleeding	6 (4.8%)
Leave small lesions	5 (4.0%)
Further tearing of posterior sheath	4 (3.2%)
Conversion to hybrid surgery	1 (0.8%)
Suture of hernial orifice	
Tearing of thread	8 (6.4%)
Minor bleeding	6 (4.8%)
Conversion to hybrid surgery	3 (2.4%)
Tearing of fascia	1 (0.8%)
Mesh placement	
Mesh too large	11 (8.8%)
Mesh too small	7 (5.6%)
Mesh not wrinkle-free	3 (2.4%)

^1^n (%)

### Technical difficulty of surgical steps

The subjective technical difficulty varied significantly across the ten standardized procedural steps (Global Kruskal-Wallis test: *p* < 0.001). Post-hoc pairwise comparisons identified Step 7 (dissection of the hernia and hernia sac) and Step 9 (closure of the anterior hernia orifice) as the most technically demanding phases of the eTEP procedure (see Table S1). Notably, no significant difference in difficulty was found between the two peak-difficulty phases, Step 7 and Step 9 (*p* = 1.000, *r* = 0.02). Step 8 (reconstruction of the posterior layer) represented a moderate technical challenge. While it was significantly more difficult than Step 4 (*p* < 0.001, *r* = 0.54), it remained significantly easier than the subsequent hernia dissection in Step 7 (*p* = 0.014, *r* = 0.39). In contrast, the initial steps of the procedure (Steps 1 through 6) showed mostly comparable difficulty levels, with no significant differences observed among them, except for Step 4, which was rated as significantly easier than most subsequent reconstructive tasks.

## Discussion

### Interpretation of the results

The main findings of the present, detailed analysis of intraoperative adverse events in eTEP show that the dissection of the hernia (step 7) and the steps involving suturing of the posterior defect or the hernia orifice (steps 8 and 9) received the highest subjective difficulty ratings. Furthermore, the results show that laparoscopic eTEP is associated with a relatively high rate of critical intraoperative events (major bleeding, injury to linea alba, conversion to open or hybrid procedure) with a cumulative incidence of 20.8%. The influence on perioperative outcomes of these critical intraoperative events are heterogenous: whereas major bleeding must be addressed immediately and injury to the linea alba should be repaired and covered with the implanted mesh, conversion to an open or hybrid procedure may be associated with less favorable postoperative outcomes, including increased postoperative pain, surgical site complications, or recurrence.

The data show that laparoscopic eTEP for ventral hernia repair may be technically challenging and appears to be associated with a relevant learning curve. A study by Montechiari et al. published in 2025 suggested that 14 cases are required to achieve procedural safety [12]. In contrast, Malcher et al. used operative time as a surrogate marker for efficacy and reported that 38 cases were necessary to reach proficiency in robotic eTEP repair [13]. The present cohort entails a small median hernia size and a relevant proportion of primary hernias, whereas in these cases, the presence of diastasis recti and/or concomitant hernias explains the choice of the laparoscopic eTEP technique. An alternative technique for those hernia types is the robotically assisted preperitoneal repair, which has been reported to yield comparable short-term outcomes to laparoscopic eTEP in selected cohorts [4]. The choice of technique in our institution depends on specialist hernia board meetings and robot availability.

In the present study, all procedures were performed exclusively by specialized hernia surgeons, which likely explains the low absolute difficulty scores during the procedural steps. The low subjective difficulty rating may underestimate technical difficulty of eTEP, but the relative difficulty of certain steps is useful to identify the most critical steps of the procedure. Despite surgical expertise, the incidence of minor and critical intraoperative events were high and likely represents daily clinical practice. Given that steps 7–9 are considered the technically most challenging in laparoscopic eTEP, the use of an on-demand robotic-assistance or the switch to total robotic eTEP repair may represent a potential strategy to facilitate technically challenging procedural steps, especially in large ventral hernias (defect size >4 cm) and in incisional hernias. Robotic assistance has been associated with improved ergonomics, enhanced visualization, and facilitated dissection and suturing, particularly in large or complex hernias and in patients with higher body mass index [14]. Robotic assistance may also lower the surgeons’ efforts, as previously seen in other studies [4]. If no robotic assistance is available, teaching through an experienced surgeon may be helpful especially during the difficult steps seven to nine.

The cumulative incidence of accidental pneumoperitoneum during the first 3 steps is 11.2%. This warrants careful surgical access and port placement at the beginning of the surgery, because accidental pneumoperitoneum during those steps may substantially increase procedural difficulty. Especially in a teaching setting, the presence of an experienced surgeon from the start of the procedure may be helpful. Accidental posterior lesions which lead to a pneumoperitoneum later during the procedure are considered normal and mostly irrelevant, as previously described by Lu et al. [15]

Furthermore, during the cross-over, an injury to the linea alba is most likely to occur (cumulative incidence of 4%) and therefore, supervision by an experienced surgeon may be beneficial. Linea alba injury may weaken the midline and predispose to an incisional hernia and we therefore recommend closure of accidental linea alba defects and assure sufficient mesh coverage. Despite the following steps with hernia dissection and suture being technically the most challenging, the likelihood of critical intraoperative events is quite low and mainly defined by the presence of an incisional hernia and the hernia diameter which may lead to conversion to a hybrid procedure, where the hernia dissection and closure is performed in an open technique, after the retromuscular space has been developed laparoscopically.

Some limitations of the presented data need mentioning. Since individual surgeons rated multiple procedures, the rating is subjective and a clustering effect may be present. The NASA-TLX response rate was 79.2%, thus an attrition bias is possible. There is no comparator group, because the study was primarily designed to assess eTEP in detail and not to compare its difficulty with other procedures. Data were pooled for primary and incisional hernias with different sizes to obtain higher case numbers. However, difficulty ratings and complexity may vary amongst those hernia entities and were not separately analyzed, which may potentially influence the ratings.

Furthermore, the median difficulty score is relatively low with 2/5, which may be an underreporting related to the procedures being carried out by specialized hernia surgeons or be related to the Likert scale of 1–5. A Likert scale of 1–10 may have led to a more detailed scattering of the difficulty scores and may have allowed greater differentiation in perceived procedural difficulty.

### Guide to the management of common and relevant pitfalls

- 1. Early pneumoperitoneum

Early pneumoperitoneum during step 1–3 may significantly impair visualization during the dissection phase and may impair continuation of the procedure. The primary guiding principle in this situation is the creation of adequate working space. As an initial measure, gas insufflation should be reduced to approximately 8 mmHg or a Verres needle or decompressing abdominal trocar may be placed. Rapid establishment of a working trocar is essential, as it allows dorsal displacement of the posterior rectus sheath and thus apply counterpressure to the upwards-pushing posterior plane. Prompt performance of the crossover is critical, as incision of the posterior rectus sheath will grant further space.

- 2. Identification of the correct level for cross-over

**Fig. 3:**
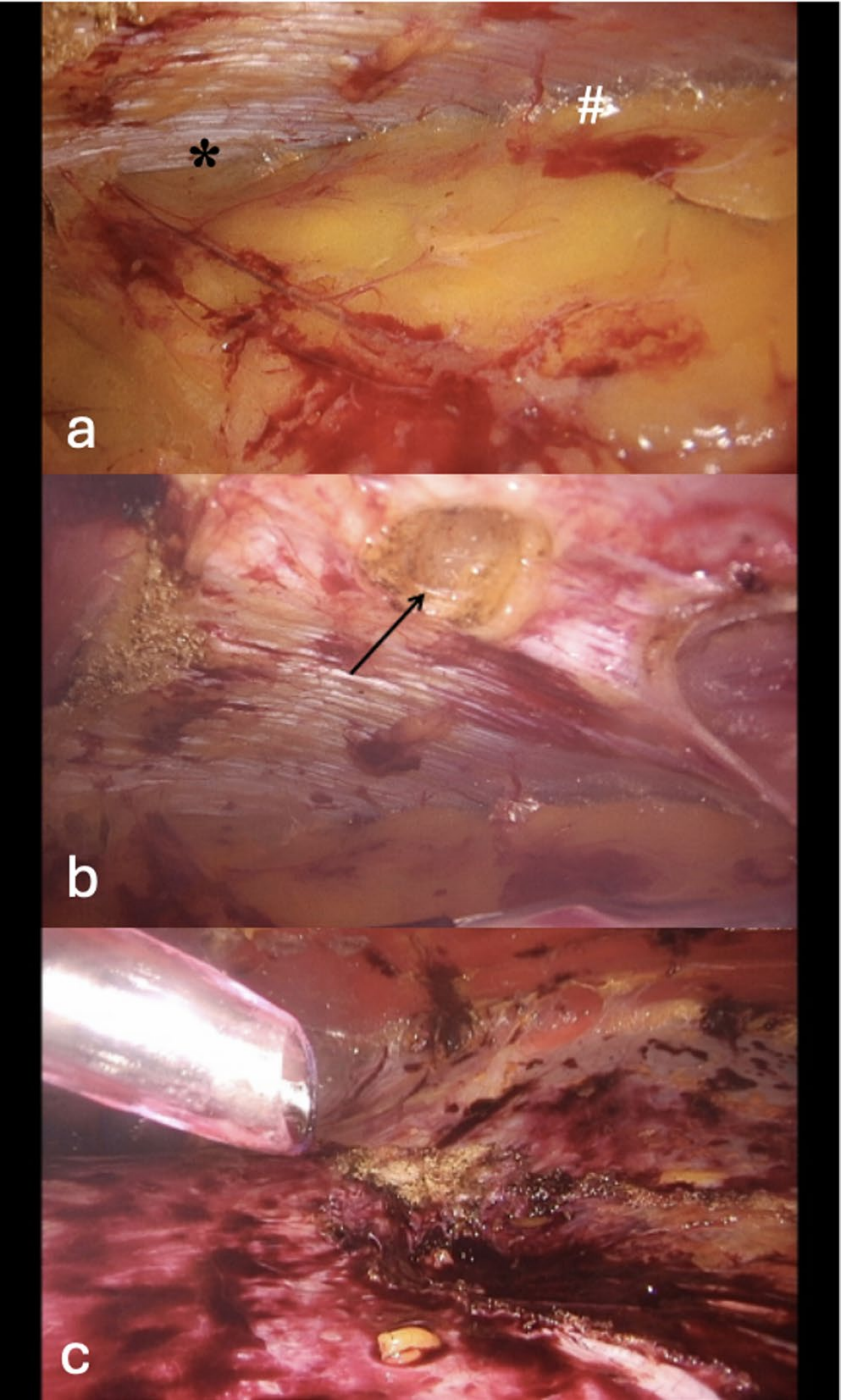
Intraoperative images in eTEP procedure. **A**: Identification of the correct level for cross-over. The presence of transverse grooves within the linea alba, in contrast to the smooth surface of the rectus sheath (*). Increased transparency of the rectus muscle fibers (#). **B**: inadvertent injury to the linea alba (black arrow). C: Sufficient mobilization of the posterior rectus sheath is indicated by the so-called “hammock sign”

After incision of the medial border, dissection is continued beneath the linea alba. Adipose tissue located inferior to the linea alba can generally be dissected without the application of excessive force. The appropriate level for crossover into the contralateral rectus sheath can be identified by the following anatomical landmarks (Fig. 3a):

- The presence of transverse grooves within the linea alba, in contrast to the smooth surface of the rectus sheath (*).
- Increased transparency of the rectus muscle fibers (#).
- Visible fasciculation of the rectus muscles following test application of monopolar electrocautery to the presumed rectus sheath.

Following incision of the rectus sheath, the rectus muscle fibers should be exposed after no more than two dissection movements. Exposure of fatty tissue alone suggests that the incision has been placed too medially, with inadvertent injury to the linea alba (see arrow in Fig. 3b). Further enlargement of this fascial opening should be avoided. Instead, a new incision should be made further lateral to the original site. Any resulting defect in the linea alba must be closed with an adequate cranial and caudal suture overlap of the lesion.

- 3. Assessment of posterior rectus sheath mobilization

Adequate, tension-free mobilization of the posterior rectus sheath is essential to prevent the development of an interparietal hernia. Sufficient mobilization is indicated by the so-called “hammock sign,” (see Fig. 3c) characterized by a concave configuration of the posterior rectus sheath that persists even under increased intra-abdominal pressure.

- 4. Inability to achieve tension-free closure of the posterior rectus sheath

If, despite adequate mobilization of the posterior rectus sheath, tension-free closure of the posterior fascial layer or peritoneal defect cannot be achieved, uni- or bilateral transversus abdominis release may be considered. If this is felt to be an overtreatment or surgical expertise for this step is not given, an alternative bailout strategy exists aside from further posterior component separation. In this situation, dissection for mesh placement should first be completed. Subsequently, a coated intraperitoneal onlay mesh should be implanted in the retromuscular position instead of a non-coated polypropylene prosthesis. The working trocars are then repositioned intra-abdominally, and the margins of the defect are secured to the mesh using fixation straps or tackers. An alternative option is the bridging of a posterior defect using a vyrcil mesh or a rotational falciform ligament flap.

- 5. Management of intraparietal hernia

Interparietal hernia is a rare but serious complication following eTEP repair. It is characterized by a defect in the posterior fascial layer with herniation of intra-abdominal viscera. Clinically, patients typically present with signs and symptoms of mechanical bowel obstruction, which may occur during the initial hospital stay or at an unplanned postoperative visit. Computed tomography is the imaging modality of choice. If clinical suspicion persists, diagnostic laparoscopy may be performed via the previous costal access to assess the bowel and the extent of the intraparietal hernia. In early cases, reduction of the hernia contents is usually feasible. The existing mesh prosthesis should be removed. Re-closure of the posterior rectus sheath is not recommended. Instead, defect coverage should be achieved using an IPOM technique, with fixation via transcutaneous sutures and additional staples. Particular attention must be paid to ensure secure fixation of the mesh along the entire defect margin.

## Conclusion

Hernia dissection and defect closure in laparoscopic eTEP seem particularly technically challenging and associated with a high surgeon workload. Given the substantial technical complexity and frequency of intraoperative adverse events observed even in expert hands, adoption of laparoscopic eTEP should occur within structured training pathways and with appropriate case selection.

## Supplementary Information

Below is the link to the electronic supplementary material.

Supplementary Material 1
