# Supplementary material for: Intraoperative adverse events and management strategies in laparoscopic enhanced-view totally extraperitoneal repair (eTEP): a guide to safe introduction
Source: Hernia. 2026 Jul 3;30(1):276. doi: 10.1007/s10029-026-03775-8 (PMC13331827; doi:10.1007/s10029-026-03775-8)
Supplement: Supplementary file 1 — Supplementary Material 1 [file 10029_2026_3775_MOESM1_ESM.pdf]

Supplementary material

Table S1: Comparative Analysis of Technical Difficulty Levels

	Step 1 (n=125)	Step 2 (n=123)	Step 3 (n=124)	Step 4 (n=122)	Step 5 (n=121)	Step 6 (n=123)	Step 7 (n=123)	Step 8 (n=91)	Step 9 (n=106)	Step 10 (n=122)
**Step 1 (n=125)**	—	0.511 (r=0.25)	1.000 (r=0.02)	1.000 (r=0.14)	1.000 (r=0.09)	0.952 (r=0.25)	**<.001 ↓ (r=0.53)**	0.051 (r=0.34)	**<.001 ↓ (r=0.61)**	1.000 (r=0.09)
**Step 2 (n=123)**	0.511 (r=0.25)	—	0.515 (r=0.24)	**0.001 ↑ (r=0.41)**	1.000 (r=0.16)	1.000 (r=0.05)	**<.001 ↓ (r=0.45)**	1.000 (r=0.17)	**<.001 ↓ (r=0.46)**	1.000 (r=0.15)
**Step 3 (n=124)**	1.000 (r=0.02)	0.515 (r=0.24)	—	0.883 (r=0.20)	1.000 (r=0.06)	0.793 (r=0.25)	**<.001 ↓ (r=0.59)**	0.058 (r=0.31)	**<.001 ↓ (r=0.62)**	1.000 (r=0.03)
**Step 4 (n=122)**	1.000 (r=0.14)	**0.001 ↓ (r=0.41)**	0.883 (r=0.20)	—	1.000 (r=0.17)	**0.003 ↓ (r=0.38)**	**<.001 ↓ (r=0.65)**	**<.001 ↓ (r=0.54)**	**<.001 ↓ (r=0.74)**	1.000 (r=0.18)
**Step 5 (n=121)**	1.000 (r=0.09)	1.000 (r=0.16)	1.000 (r=0.06)	1.000 (r=0.17)	—	1.000 (r=0.18)	**<.001 ↓ (r=0.54)**	0.511 (r=0.26)	**<.001 ↓ (r=0.56)**	1.000 (r=0.02)
**Step 6 (n=123)**	0.952 (r=0.25)	1.000 (r=0.05)	0.793 (r=0.25)	**0.003 ↑ (r=0.38)**	1.000 (r=0.18)	—	**<.001 ↓ (r=0.49)**	1.000 (r=0.12)	**<.001 ↓ (r=0.45)**	1.000 (r=0.18)
**Step 7 (n=123)**	**<.001 ↑ (r=0.53)**	**<.001 ↑ (r=0.45)**	**<.001 ↑ (r=0.59)**	**<.001 ↑ (r=0.65)**	**<.001 ↑ (r=0.54)**	**<.001 ↑ (r=0.49)**	—	**0.014 ↑ (r=0.39)**	1.000 (r=0.02)	**<.001 ↑ (r=0.54)**
**Step 8 (n=91)**	0.051 (r=0.34)	1.000 (r=0.17)	0.058 (r=0.31)	**<.001 ↑ (r=0.54)**	0.511 (r=0.26)	1.000 (r=0.12)	**0.014 ↓ (r=0.39)**	—	0.512 (r=0.32)	0.512 (r=0.29)
**Step 9 (n=106)**	**<.001 ↑ (r=0.61)**	**<.001 ↑ (r=0.46)**	**<.001 ↑ (r=0.62)**	**<.001 ↑ (r=0.74)**	**<.001 ↑ (r=0.56)**	**<.001 ↑ (r=0.45)**	1.000 (r=0.02)	0.512 (r=0.32)	—	**<.001 ↑ (r=0.59)**
**Step 10 (n=122)**	1.000 (r=0.09)	1.000 (r=0.15)	1.000 (r=0.03)	1.000 (r=0.18)	1.000 (r=0.02)	1.000 (r=0.18)	**<.001 ↓ (r=0.54)**	0.512 (r=0.29)	**<.001 ↓ (r=0.59)**	—

Surgical Step Definitions:
Step 1: Initial access and placement of the first trocar; Step 2: Dissection of the retrorectus space on the access side; Step 3: Establishment of the triangulation / working trocars; Step 4: Medial incision of the posterior rectus sheath (access side); Step 5: Crossover and incision of the contralateral posterior rectus sheath; Step 6: Dissection of the contralateral retrorectus space; Step 7: Dissection of the umbilical/incisional hernia and hernia sac; Step 8: Reconstruction and suturing of the posterior layer; Step 9: Direct closure and suturing of the anterior hernial orifice; Step 10: Final positioning and unfolding of the sublay mesh

Interpretation of Table Cells:
Cells display the adjusted p-value and the **Effect Size (*r*)** in parentheses. **Red/Bold** text indicates statistical significance ($p < 0.05$). The arrows (↑ / ↓) describe the relative difficulty of the step in the **vertical row** compared to the step in the **horizontal column header**: An **upward arrow (↑)** signifies that the step in the row was significantly more difficult (higher rank) than the step in the column. A **downward arrow (↓)** indicates the row step was significantly easier.

Effect Size (*r*) Classification:
The effect size *r* is calculated as $Z/\sqrt{N}$. Following Cohen's criteria, values are interpreted as: ~0.10: Small effect (minimal clinical relevance); ~0.30: Medium effect (noticeable clinical relevance); ≥0.50: Large effect (major clinical impact).

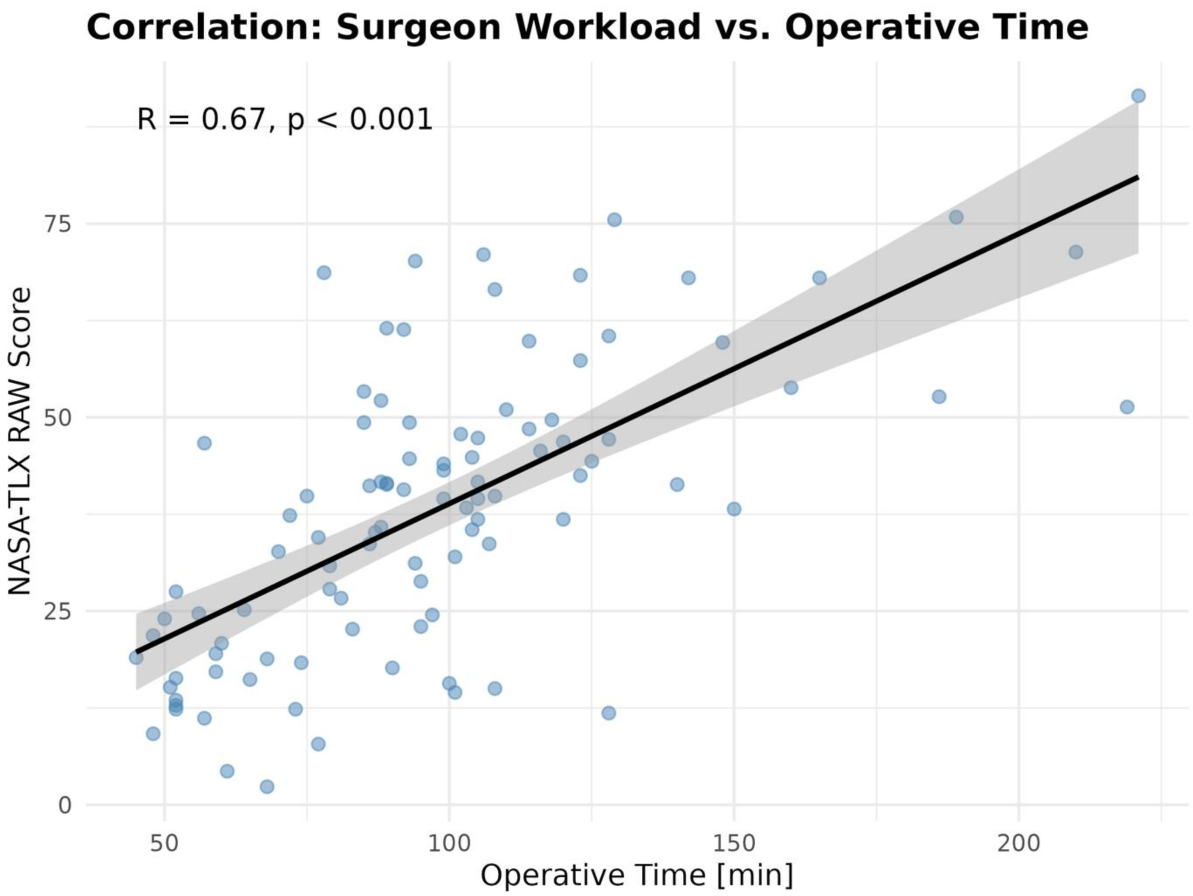

Figure S1: Correlation between Surgeon workload using NASA-TLX and operative time.
